# Association Between Maternal Hyperglycemia and Composite Maternal-Birth Outcomes

**DOI:** 10.3389/fendo.2018.00755

**Published:** 2018-12-11

**Authors:** Song-Ying Shen, Li-Fang Zhang, Jian-Rong He, Jin-Hua Lu, Nian-Nian Chen, Wan-Qing Xiao, Ming-Yang Yuan, Hui-Min Xia, Kin Bong Hubert Lam, Xiu Qiu

**Affiliations:** ^1^Division of Birth Cohort Study, Guangzhou Women and Children's Medical Center, Guangzhou Medical University, Guangzhou, China; ^2^Nuffield Department of Population Health, University of Oxford, Oxford, United Kingdom

**Keywords:** hyperglycemia, pregnancy, stillbirth, birth weight, duration of pregnancy, preeclampsia, cesarean section, composite outcome

## Abstract

**Objective:** The overall impact of maternal hyperglycemia on maternal and birth outcomes is largely underestimated, therefore quantifying the true burden of hyperglycemia in a whole population it is a challenging task. This study aims at examining the association between blood glucose concentration during pregnancy and a composite score of adverse maternal-birth outcomes in a large-scale prospective cohort study in China.

**Methods:** Pregnant women within “the Born in Guangzhou Cohort Study” China who underwent a standard 75-g oral-glucose-tolerance-test (OGTT) between 22 and 28 gestational weeks were included. A composite score of stillbirth, duration of pregnancy, birth weight, preeclampsia, and cesarean section was developed based on a published maternal-fetal outcomes scale, weighed by the relative severity of the outcomes. Multiple linear regression models were used to assess the associations between OGTT glucose measurements and log composite score. Logistic regression models were used to assess relations with outcome as a categorical variable (0, 1– < 3, and ≥3).

**Findings:** Among 12,129 pregnancies, the composite score ranged from 0 to 100 with a median of 2.5 for non-zero values. Elevated fasting glucose level was associated with higher composite score (adjusted coefficients 0.03 [95% CI, 0.02–0.04] for 1-SD increase). For 1-SD increase in fasting glucose, the risk of having a composite score 1– < 3 and ≥3 rises by 13% (95% CI, 8–17%) and 15% (95% CI, 7–23%), respectively. Similar association and increase in risk was found for 1 and 2-h glucose.

**Conclusion:** Elevated fasting, 1 and 2-h glucose levels are associated with a range of adverse maternal-birth outcomes. The composite score model can be applied to the risk assessment for individual pregnant women and to evaluate the benefits for controlling glucose levels in the population.

## Introduction

Gestational diabetes mellitus (GDM) is one of the most prevalent major complications during pregnancy worldwide (1–3). Previous studies have linked maternal hyperglycemia to increased risks of various adverse perinatal outcomes, including macrosomia, large for gestational age (LGA), cesarean section, preterm birth, gestational hypertension and hyperbilirubinemia (3, 4). However, all such studies have only investigated the perinatal outcomes in isolation without taking into account the complex inter-relationship between maternal and fetal outcomes, and among the perinatal outcomes themselves; examples include macrosomia and cesarean section (5), gestational hypertension and preterm birth (6, 7). In addition, the varying strengths of association with these outcomes, which have different levels of severity and consequences, have not been fully appreciated (3, 4, 8). This has led to difficulties in assessing the influence of maternal hyperglycemia on both maternal and birth outcomes, and developing optimal management of maternal hyperglycemia, as well as evaluating the effectiveness of glucose-lowering interventions in the general pregnant women population. A comprehensive consideration of multiple adverse outcomes, both in pregnant women and in newborns (9), is therefore needed.

Indeed, some previous studies have attempted to assess multiple perinatal outcomes (8–12). For example, to evaluate the need of care during pregnancy and delivery, Novicoff et al. created an outcome score that sums the points of all maternal and fetal outcomes based on relative desirability and frequency of occurrence (10). Verma et al. developed a morbidity assessment index for newborns (called MAIN score), capturing the entire severity spectrum of morbidity at birth to estimate the effectiveness of obstetric interventions on neonatal morbidity (9, 12). However, none of these studies have evaluated the influence of maternal hyperglycemia on composite maternal and birth outcomes. A comprehensive measure focusing on capturing GDM-related morbidity in both mother and child should add an important dimension to current understanding of GDM risk and management.

The present study aims to examine the association between blood glucose concentrations during pregnancy, and risk-adjusted adverse maternal-birth outcomes represented by a composite score in a large-scale prospective cohort study in China.

## Materials and Methods

### Study Design and Population

**Figure 1:**
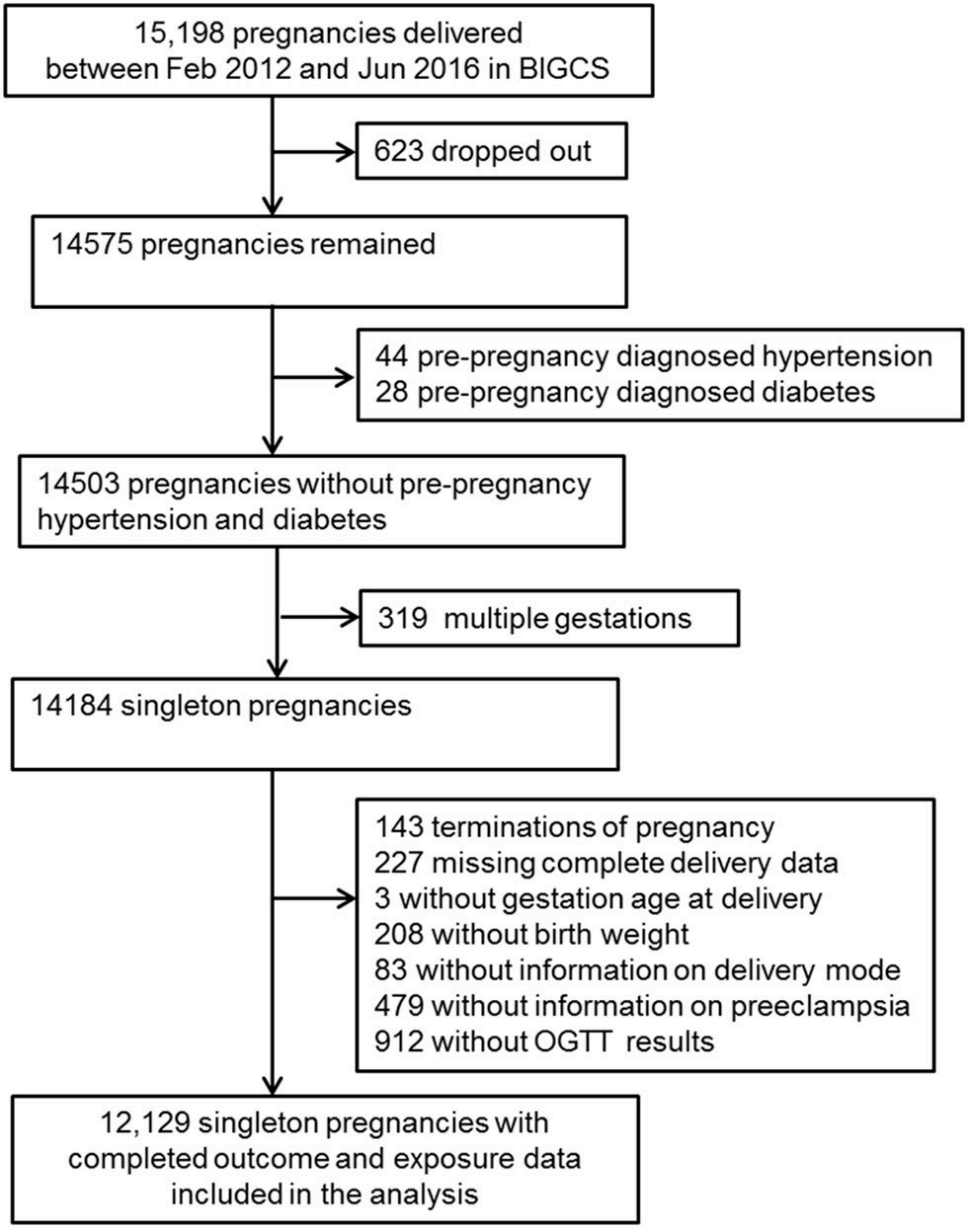
The flowchart of the study.

The data used in the present study is part of the Born in Guangzhou Cohort Study (BIGCS), which is a birth cohort study conducted in the Guangzhou Women and Children's Medical Center (GWCMC), China. Study participants were recruited from pregnant women attending their first routine antenatal exam at GWCMC. Inclusion criteria were: residents in Guangzhou, <20 weeks gestation, intending to deliver at GWCMC, and planning to remain in Guangzhou with the child for at least 3 years after delivery. A detailed description of the BIGCS protocol has been published elsewhere (13). For this analysis, we excluded participants with pre-pregnancy diabetes or chronic hypertension, according to the self-reported medical history. Women with missing blood glucose and delivery data were also excluded (Figure 1).

At enrollment participants underwent an interviewer-administered questionnaire, which collected a wide range of information including socio-demographic data, exposures at home and at workplace, personal lifestyle, medical histories, and health status before pregnancy. The study protocol was approved by GWCMC Ethics Approval Board. Written informed consent was obtained from all participants.

### Oral Glucose-Tolerance Test

Between 22 and 28 weeks of gestation, all pregnant women underwent a standard oral glucose tolerance test (OGTT), during which blood samples (2 mL) were collected at fasting, 1 and 2 h after a 75 g glucose load with NaF/EDTA tubes. Detailed procedures of the OGTT test were presented elsewhere (14). Women with GDM diagnosis [based on the International Association of Diabetes and Pregnancy Study Groups [IADPSG] criteria (15)] received routine consultation on diet and exercise. They were then asked to self-monitor their preprandial blood glucose levels after having dietary management for 3–5 days. Those with fasting blood glucose ≥5.3 mmol/L or 2 h postprandial glucose ≥6.7 mmol/L after dietary control were prescribed insulin in addition to a diet and exercise regime (16).

### Maternal and Birth Outcomes

Information about pregnancy complications, mode of delivery, gestational age, birth weight, parity, and gender of newborns were obtained from GWCMC's electronic medical records. Gestational age was confirmed by ultrasound examination in the first- or second-trimester. Birth weight was measured by midwives immediately after delivery. Birthweight z scores were calculated using a local population-based birth weight reference (17). LGA was defined as a birthweight larger than the 90th percentile for gestational age by gender and small for gestational age (SGA) was defined as a birthweight lower than the 10th percentile for gestational age by gender, based on the same birth weight reference (17). Preterm birth was defined as birth before 37 weeks of gestation. Spontaneous preterm birth was the birth following spontaneous preterm labor and/or preterm premature rupture of the membranes before 37 weeks of gestation, irrespective of the mode of delivery (vaginal, cesarean section) (18); all this information was obtained from medical records, which was also independently confirmed by two pediatricians. Gestational hypertension was defined as blood pressure ≥140/90 mmHg on at least two occasions separated by at least 4 h after 20th week of gestation without the presence of protein in the urine and returned to normal within 12 weeks postpartum (19). Preeclampsia was defined as gestational hypertension combined with proteinuria [protein level in the urine ≥300 mg/24 h or ≥(+) by a dipstick test on at least two occasions separated by at least 6 h] (19). Stillbirth cases were identified from the medical records with the 10th revision of the International Classification of Diseases (ICD10) codes of O36.401 and P95.

We developed a composite outcome score based on the adverse maternal and infant outcomes related to GDM as suggested by the literature (3, 4); maternal-infant outcomes included stillbirth, duration of pregnancy, birth weight, preeclampsia, and cesarean section. A score was assigned to each outcome according to a published unified maternal-fetal outcome risk assessment model, ranging from 0 (perfect outcome) to 100 (maternal or infant death) (10). Each mother-newborn pair was scored based on the existence and severity of the five outcomes as shown in Table 1. A comprehensive outcome score was calculated by summing all above-mentioned outcomes scores for each mother-newborn pair. Higher scores indicated the presence of worse maternal and perinatal outcomes.

**Table 1:** Maternal-birth outcomes scoring scale.

**Outcomes**	Score^*^
Stillbirth	100
Preeclampsia	5
Cesarean delivery	2.5
Gestational age ≤ 24 wk	11
Gestational age 25–26 wk	7
Gestational age 27–28 wk	6
Gestational age 29–30 wk	4
Gestational age 31–33 wk	3
Gestational age 34–36 wk	2
Gestational age ≥37 wk and < 42 wk	0
Gestational age >42 wk	1
Birth weight < 750 g	10
Birth weight 750–1,000 g	7
Birth weight 1,000–1,500 g	5
Birth weight 1,500–2,500 g	3
Birth weight 2,500–4,000 g	0
Birth weight >4,000 g	1

* *The score assigned to each outcome was referred to a published unified maternal-fetal outcome risk assessment model, ranging from 0 (perfect outcome) to 100 (maternal or infant death) (10)*.

### Potential Confounders

The following maternal risk factors were selected a priori as potential confounders: age, maternal education level, maternal income, pre-pregnancy body mass index (BMI), maternal smoking status, secondhand smoking (smoke exposure during pregnancy), use of assisted reproduction technology, parity, and gestational age at the time of OGTT. Except for gestational age at OGTT and parity, all information was derived from the self-reported questionnaire at enrollment.

### Statistical Analysis

Descriptive statistics of the characteristics and outcomes were reported for the whole sample and across categories of the composite scores. To assess the relationship between OGTT glucose measurements and maternal and fetal outcomes as represented by the composite score, we analyzed the data in two ways. First, the composite score was treated as a continuous variable and was log (base 10) transformed [log (score+1)]. Multiple linear regression models were used to assess the association between the OGTT glucose measurements and log composite score. Regression coefficients were calculated, representing the change in log composite score for 1-SD change in each fasting, 1 and 2 h glucose measurement. Second, we categorized the composite scores (without transformation) into three groups based on the median of non-zero scores (2.5): 0, low (including scores 1, 2, and 2.5) and high (≥3). Logistic regression models were used to evaluate the relationships of the OGTT glucose measurements with composite outcomes score categories, with “0” as reference. Odds ratios (ORs) were calculated for a 1-SD increase in each OGTT measurement. The linear and logistic regression models were adjusted for pre-specified potential confounders, including maternal age (continuous), maternal education (middle school or below, college, undergraduate or postgraduate), maternal income (< 1,500, 1,500–4,500, 4,501–9,000, ≥9,001 Yuan), maternal pre-pregnancy body mass index (BMI, continuous), parity (0, ≥1), smoking status (yes, no), second-hand smoking exposure during pregnancy (yes, no), use of assisted reproduction technology (yes, no), and gestational age when OGTT was performed (continuous).

All analyses were performed using SAS 9.3 software (SAS Institute, Cary, NC, USA). *P* < 0.05 were considered to indicate statistical significance.

## Results

A total of 15,198 pregnant women in BIGCS delivered between February 2012 and January 2016. We excluded women with multiple births, those who dropped out before delivery or terminated their pregnancy, had pre-pregnancy diagnosed hypertension and diabetes, or whose delivery data and OGTT results were missing, resulting in 12,129 mothers and their singleton births in this analysis (Figure 1).

Characteristics of mothers and newborns, and by composite score groups are shown in Table 2. Pregnant women had a mean age of 29.1 years (SD 3.4). The majority of them had attained college or above qualifications and over half of them had a monthly income more than 4,500 Yuan. Few women smoked but 30% were exposed to secondhand smoke during pregnancy. Not unexpectedly, the vast majority (86.6%) of the pregnant women were primipara. Only 3.4% women undertook assisted reproduction technology. About 10% of the participants were overweight or obese before pregnancy BMI ≥24.0 kg/m^2^, according to the Chinese guidelines (20). The mean plasma glucose levels were 4.3 mmol/L (SD 0.4) at fasting, 7.7 mmol/L (SD 1.7) at 1-h, and 6.6 mmol/L (SD 1.3) at 2-h post 75 g glucose load, respectively. Using the IADPSG criteria, there were 1,662 (1,662/1,662, 100%) women diagnosed with GDM who received a subsequent diet and exercise advice. Ten (10/1,662, 0.6%) women went on to receive insulin therapy. Around 60% of the pregnancies (*n* = 7,296) resulted in optimal outcome (with a score of 0), one-third (*n* = 3,935) having scores between 1 and < 3, and 7% (*n* = 898) had a composite score ≥3 (20% being 3). There were statistically significant differences in most of the maternal characteristics measured across the three composite score groups.

**Table 2:** Characteristics of the 12,129 study participants and their newborns by different groups of composite score.

**Characteristic**	**All participants**	**Composite score**			
		**0**	**1– <3**	**≥3**	***p*-value**
*n*	12,129	7,296	3,935	898
Age (years), mean ± SD	29.1 ± 3.4	28.6 ± 3.1	29.9 ± 3.7	29.5 ± 3.6	< 0.0001
**PLASMA GLUCOSE (mmol/L), MEAN ± SD**					
Fasting	4.3 ± 0.4	4.3 ± 0.4	4.3 ± 0.4	4.3 ± 0.5	< 0.0001
1-h	7.7 ± 1.7	7.6 ± 1.6	7.8 ± 1.7	7.9 ± 1.8	< 0.0001
2-h	6.6 ± 1.3	6.5 ± 1.3	6.8 ± 1.4	6.8 ± 1.5	< 0.0001
Length of gestation at time of OGTT (wk), mean ± SD	24.5 ± 1.6	24.5 ± 1.6	24.5 ± 1.6	24.4 ± 1.6	0.441
Income (Yuan), *n* (%)					<0.0001
< 1,500	1,115 (9.6)	674 (9.7)	363 (9.7)	78 (9.3)
1,500–4,500	3,460 (29.9)	2,207 (31.6)	1,001 (26.7)	255 (30.4)
4,501–9,000	4,947 (42.8)	2,933 (42.0)	1,641 (43.8)	373 (44.5)
≥9,001	2,040 (17.7)	1,166 (16.7)	741 (19.8)	133 (15.9)
Education, *n* (%)					0.0051
Middle school or below	1,112 (9.2)	645 (8.8)	377 (9.6)	90 (10.0)
College	3,068 (25.3)	1,847 (25.3)	969 (24.6)	252 (28.1)
Undergraduate	6,472 (53.4)	3,929 (53.9)	2,067 (52.5)	476 (53.0)
Postgraduate	1,471 (12.2)	875 (12.0)	522 (13.3)	80 (8.9)
Pre-pregnancy body mass index (kg/m^2^), mean ± SD and *n* (%)	20.4 ± 2.7	20.1 ± 2.5	20.8 ± 2.8	20.7 ± 3.0	< 0.0001
≤ 18.5	2,967 (25.0)	1,994 (27.9)	768 (20.0)	205 (23.6)	< 0.0001
18.5–23.9	7,785 (65.6)	4,634 (64.8)	2,598 (67.5)	553 (63.6)
24.0–27.9	939 (7.9)	450 (6.3)	398 (10.3)	91 (10.5)
≥28.0	181 (1.5)	74 (1.0)	86 (2.2)	21 (2.4)
Smoking during pregnancy, *n* (%)	59 (0.5)	34 (0.5)	16 (0.4)	9 (1.0)	0.0584
Passive smoking during pregnancy, *n* (%)	3,494 (29.6)	2,166 (30.4)	1,082 (28.3)	246 (28.5)	0.0467
Parity at enrollment ≥1, *n* (%)	1,613 (13.4)	845 (11.7)	654 (16.8)	114 (12.8)	< 0.001
Use of assisted reproduction technology, *n* (%)	396 (3.4)	182 (2.6)	168 (4.4)	46 (5.3)	< 0.0001

*OGTT, oral glucose tolerance test*.

Overall, the prevalence of preeclampsia (0.7%) and stillbirth (0.1%) was low, although one-third of deliveries were by cesarean section. Infants were born weighing on average 3191.5 g (SD 423.9) after a mean gestational length of 38.8 weeks (SD 1.4), with 3.5% of the babies having spontaneous preterm birth (< 37 weeks).

Among those who had a non-zero composite score, 4,138 (84.2%) had one outcome, 620 (12.6%) had two, 155 (3.2%) had three or more. Cesarean delivery was the most common outcome, occurring in 4,161 (85.0%) of the women with non-zero score.

Table 3 summarizes selected pregnancy-related and birth outcomes by composite score groups. The prevalence of gestational hypertension, preeclampsia, cesarean delivery, preterm birth, spontaneous preterm birth, LGA, SGA, and stillbirth increased significantly with higher composite score categories.

**Table 3:** Obstetrical and newborn outcomes of the 12,129 study participants.

**Characteristic or outcome**	**All participants**	**Composite score**		
		**0**	**1– < 3**	**≥3**
*n*	12,129	7,296	3,935	898
**OBSTETRICAL OUTCOMES**				
Hypertension, *n* (%)	295 (2.4)	82 (1.1)	100 (2.5)	113 (12.8)
Gestational hypertension	214 (1.8)	82 (1.1)	100 (2.5)	32 (3.6)
Preeclampsia	81 (0.7)	–	–	81 (9.15)
Cesarean delivery, *n* (%)	4,161 (34.3)	–	3,639 (92.5)	522 (59.0)
**NEWBORN OUTCOMES**				
Stillbirth, *n* (%)	13 (0.1)	–	–	13 (1.4)
Gestational age at delivery (week), mean ± SD	38.8 ± 1.4	39.0 ± 1.0	38.9 ± 1.2	36.7 ± 2.7
Preterm birth, *n* (%)	579 (4.8)	–	188 (4.8)	391 (44.5)
Spontaneous preterm birth, n (%)	419 (3.5)	–	169 (4.3)	250 (33.9)
Birth weight (g), mean ± SD	3191.5 ± 423.9	3194.5 ± 316.8	3292.0 ± 373.8	2720.5 ± 863.5
Birth weight Z-score, mean ± SD	0.1 ± 1.0	0.0 ± 0.8	0.3 ± 0.9	−0.2 ± 1.8
**BIRTH WEIGHT FOR GESTATIONAL AGE**, ***N*** **(%)**				
SGA	875 (7.3)	431 (5.9)	122 (3.1)	322 (36.7)
AGA	9,917 (82.3)	6,354 (87.6)	3,210 (81.9)	353 (40.2)
LGA	1,254 (10.4)	465 (6.4)	586 (15.0)	203 (23.1)

*SGA, small for gestational age, AGA, appropriate for gestational age, LGA, large for gestational age*.

Table 4 shows the associations between fasting, 1 and 2-h plasma glucose and composite perinatal outcomes score after adjusted for confounders. Elevated fasting glucose values was significantly associated with higher log composite score as a continuous variable (coefficient 0.03 [95% CI, 0.02–0.04] for 1-SD increase). For 1-SD increase in fasting glucose, the likelihoods of having composite outcomes score 1– < 3 and ≥3 increased by 13% (95% CI, 8–17%) and 15% (95% CI, 7–23%) in the logistic regression model, respectively. Similar associations were found for 1 and 2-h glucose values.

**Table 4:** Adjusted coefficients and odds ratios for associations between maternal glycemia and composite maternal-birth outcomes.

**Composite score**	**Plasma glucose level**						
		**Fasting**		**At 1-h**		**At 2-h**	
	***n***	**Crude**	**Adjusted^a^**	**Crude**	**Adjusted^a^**	**Crude**	**Adjusted^a^**
Continuous, coefficients (95% CI)^b^	12,129	0.05 (0.04, 0.07)	0.03 (0.02, 0.04)	0.05 (0.04, 0.06)	0.02 (0.01, 0.04)	0.06 (0.05, 0.07)	0.03 (0.02, 0.04)
**CATEGORICAL, OR (95% CI)**							
0	7,296	Ref.	Ref.	Ref.	Ref.	Ref.	Ref.
1- < 3	3,935	1.20 (1.15, 1.25)	1.13 (1.08, 1.17)	1.16 (1.12, 1.21)	1.06 (1.02, 1.11)	1.19 (1.15, 1.24)	1.09 (1.05, 1.14)
≥3	898	1.21 (1.13, 1.29)	1.15 (1.07, 1.23)	1.22 (1.14, 1.30)	1.15 (1.07, 1.24)	1.22 (1.14, 1.30)	1.14 (1.06, 1.22)

*CI, confidence interval, OR, odd ratio*.

a *Regression coefficients or odds ratios were for an increase in the glucose level of 1 SD (0.42 mmol/L for the fasting glucose level, 1.66 mmol /L for the 1 h glucose level, and 1.35 mmol/L for the 2 h glucose level.). Adjusted for age, income, educational level, smoking during pregnancy, second hand smoking exposure during pregnancy, pre-pregnancy BMI, parity, assisted reproductive technology, gestational age at the OGTT*.

b *Composite score was log transformed as log(score+1)*.

## Discussion

In the present study, a composite maternal-birth outcome scoring scale based on five perinatal outcomes that are most related to gestational diabetes was adapted from a risk model covering maternal and birth outcomes. Significant associations between increased fasting, 1 and 2-h post-load plasma glucose levels and increased composite maternal and birth outcomes score were found.

A number of studies have investigated the associations between maternal glucose levels and individual perinatal outcomes, such as preterm birth or LGA (3, 4). However, none has considered the multiple adverse maternal and birth outcomes holistically in the same study. Individual outcomes, e.g., LGA, cesarean delivery, preterm birth, preeclampsia, when considered separately, are unable to capture the overall impact of maternal hyperglycemia in mothers and their newborns, hampering the risk assessment of maternal hyperglycemia and the evaluation of effectiveness and benefit for glucose level management in the general pregnant women population. In the present study, the composite outcome score included birth weight and gestational age, both of which have been widely used as outcome measures of effectiveness of national health policies and interventions for pregnant women, especially in developing countries (17, 18), and have been reported to be strongly associated with GDM (via abnormal birthweight and preterm birth) (4, 21). We also included preeclampsia and cesarean section—both associated with elevated glucose concentrations during pregnancy (4, 21). Hence, the composite score combining complications in both mother and newborn is an interpretable measure of the actual morbidity that are most related to maternal hyperglycemia during pregnancy.

In the present analysis, we found that the risk of having a higher composite score (between 1 and < 3, ≥3) decreased for 1-SD lower in fasting, 1 and 2-h glucose levels. This may help to assess the risk of developing adverse maternal and birth outcomes for individual pregnant women and recognize specific needs for hyperglycemic women, thus facilitating resource-allocation and care optimization for the high-risk, high-cost patient encounter (8, 9). For example, among the women with hyperglycemia, only those who have high risk of overall adverse outcomes need more intensive care and intervention. On the other hand, the linear association between maternal glucose level and the comprehensive outcome score highlights the importance of controlling maternal plasma glucose at low levels, rather than aiming at the level just below the diagnostic cut-off. While there is evidence of substantial benefits from intervention for women with GDM and their newborns, previous intervention studies reported improvement in targeted morbidities separately (22, 23) rather than across a spectrum of major morbidities to assess overall health improvement. As a result, clinicians may opt to implement different treatment and management strategies depending on the particular outcome they focus on, which may not necessarily be the best option for the patients. The results also can be applied to evaluate the overall effectiveness and benefit of controlling glucose level in the whole pregnant women population. According to our findings, if the fasting glucose level increases by 1-SD (0.42 mmol/L) in pregnant women, the risk of having a composite adverse outcomes score 1- < 3 and ≥3 would increase by 13 and 15%, respectively. Consider China where there is a large number of pregnant women, ~18 million per year, an excess of 0.76 million (13% × 32.4% [prevalence of women with composite score 1– < 3] × 18 million) and 0.20 million (15% × 7.4% [prevalence of women with composite score ≥3] × 18 million) women per year would suffer from mild and relative severe adverse maternal and birth outcomes if fasting glucose would increase by 1-SD in the population. Adequate control of fasting glucose concentration is therefore necessary in both individual and population levels.

The strengths of our study include the use of high-quality outcome data extracted from medical records and that a wide range of potential confounders have been considered in the analysis. This study had some limitations though. First, we did not adjust for the dietary intake of the women during pregnancy, which could potentially affect fetal growth and other maternal-birth outcomes. Second, we recognize that the weights we applied for the five outcomes in the present study, adapted from the maternal and infant scoring system developed by Novicoff et al. (10), may not accurately reflect the relative severity of the outcomes. However, there has been no universally agreed standard values and ranking for the outcomes of interest. Once a more appropriate standardized scale for each outcome is developed, studies like ours will be required to confirm the findings. Third, neonatal hypoglycaemia is also an important outcome related to gestational diabetes. Unfortunately, the scale we used did not include hypoglycaemia as an outcome and we also did not collect data about this outcome.

In conclusion, using a composite scale that ranks the relative severity of multiple maternal and infant outcomes, we assessed the effects of elevated fasting, 1 and 2-h post-load plasma glucose and confirmed the potential benefits of adequate control of maternal glucose level in improving maternal-birth outcomes as a whole. This could be applied to the risk assessment for individual pregnant women as well as to the evaluation of effectiveness and benefit for controlling glucose level in the population.

## Author Contributions

XQ and H-MX designed the study and directed its implementation. S-YS, J-RH, J-HL, N-NC, W-QX, and M-YY were involved in study design, questionnaires development, data collection, and follow-up of participants. S-YS participated in the data analysis and drafted the manuscript. L-FZ carried out the data analysis, contributed to the writing of the paper. J-HL managed the data. KL and XQ revised the manuscript. All authors critically revised the manuscript, and approved the final version.

### Conflict of Interest Statement

The authors declare that the research was conducted in the absence of any commercial or financial relationships that could be construed as a potential conflict of interest.
